# Advances in the combination of CAR-T therapy with small-molecule reagents for hematologic malignancies

**DOI:** 10.3389/fimmu.2025.1663522

**Published:** 2025-10-15

**Authors:** Baiyan Yang, Yang Su, Chunling Wang, Liang Yu

**Affiliations:** ^1^ Department of Hematology, The Affiliated Huaian No.1 People’s Hospital of Nanjing Medical University, Huaian, China; ^2^ Department of Hematology, Key Laboratory of Autoimmune Diseases of Huaian City, Huaian, China

**Keywords:** CAR-T cells, small-molecule reagents, hematologic malignancies, combination therapy, safety

## Abstract

Chimeric antigen receptor T-cell (CAR-T) therapy has become a cornerstone of the treatment for hematologic malignancies. However, as a monotherapy, it still faces major challenges such as therapy resistance, disease relapse, and minimal residual disease (MRD) persistence post-remission. Small molecule compounds, such as Bruton’s tyrosine kinase (BTK) inhibitors, hypomethylating agents, and immunomodulatory drugs, can augment the antitumor efficacy of CAR-T cells through multiple mechanisms, enhancing their persistence and the proliferative capacity. Combination therapy enables a synergistic attack on malignant cells to effectively eliminate MRD, overcome resistance, and improve overall therapeutic outcomes. To date, the combining of CAR-T therapy with small molecule agents has shown promising clinical potential in the treatment of hematologic malignancies, including acute lymphoblastic leukemia (ALL), diffuse large B-cell lymphoma (DLBCL), chronic lymphocytic leukemia (CLL) and multiple myeloma (MM). This review provides a comprehensive overview of the efficacy, safety and synergistic mechanisms of CAR-T therapy combined with Small-molecule reagents, and proposes optimization strategies to enhance clinical efficacy and minimize the adverse effects which may pave the way for future therapeutic development.

## 1 Introduction

Chimeric antigen receptor T cells (CAR-T) are autologous T lymphocytes that have been genetically engineered to express a CAR, consisting of an extracellular antigen recognition domain linked to intracellular co-stimulatory signaling motifs. This synthetic receptor architecture enables the precise recognition of tumor-associated antigens, eliciting potent cytotoxic immune responses. CAR-T therapy has been recognized as a transformative intervention, demonstrating remarkable clinical efficacy. Clinical studies, such as the pivotal JULIET trial (NCT02445248), have demonstrated that CAR-T monotherapy induces complete remission (CR) in approximately 40% of patients with relapsed/refractory diffuse large B-cell lymphoma (R/R DLBCL) (1). Currently, CD19-targeted CAR-T therapies have been approved by the U.S. Food and Drug Administration (FDA) as monotherapy for treating relapsed/refractory aggressive B-cell lymphomas and indolent B cell lymphomas. Despite CAR-T therapy becoming a cornerstone in the treatment of hematologic malignancies, monotherapy still face significant challenges. These including the limited *in vivo* persistence of CAR-T cells (2), immunosuppression within the tumor microenvironment (TME) (3), off-target toxicities, antigen loss and the development of acquired resistance mechanisms (4). Findings from the JULIET and TRANSCEND NHL-001 trials indicate that approximately 27%–47% of patients with R/R DLBCL exhibit primary resistance to CD19-directed CAR-T therapy (5, 6), and 30–50% relapse after initial remission (7). Furthermore, cytokine release syndrome (CRS) occurred in 42%–58% of patients following CD19-directed CAR-T therapy for B-cell malignancies, primarily driven by CAR-T activation and macrophage-mediated cytokine release. And immune effector cell-associated neurotoxicity syndrome (ICANS) was reported in 20%–30% of patients; although its precise pathophysiology remains unclear, it is generally linked to blood–brain barrier disruption, endothelial activation, and IL-1–mediated neuroinflammation (5, 6). To increase the effectiveness of CAR-T therapy and overcome its limitations, combination strategies involving CAR-T cells and small molecule reagents have emerged as a promising investigational approach. Preclinical and clinical evidence suggests that Small-molecule reagents may enhance CAR-T cell functionality by promoting the activation (8), prolonging *in vivo* persistence, reprogramming the immunosuppressive tumor microenvironment (iTME), and counteracting intrinsic or acquired resistance mechanisms, thereby improving the objective response rates (ORR) and durable remission rates in these patients. This review comprehensively examines the mechanistic underpinnings, clinical advances, efficacy and safety profiles of CAR-T therapy combined with Small-molecule reagents in the treatment of hematologic malignancies. Additionally, it highlights emerging strategies for future development of these combination regimens, with the aim of providing scientific evidence to guide their clinical translation.

## 2 Mechanisms underlying the synergistic effects of CAR-T therapy and small-molecule reagents

**Figure 1:**
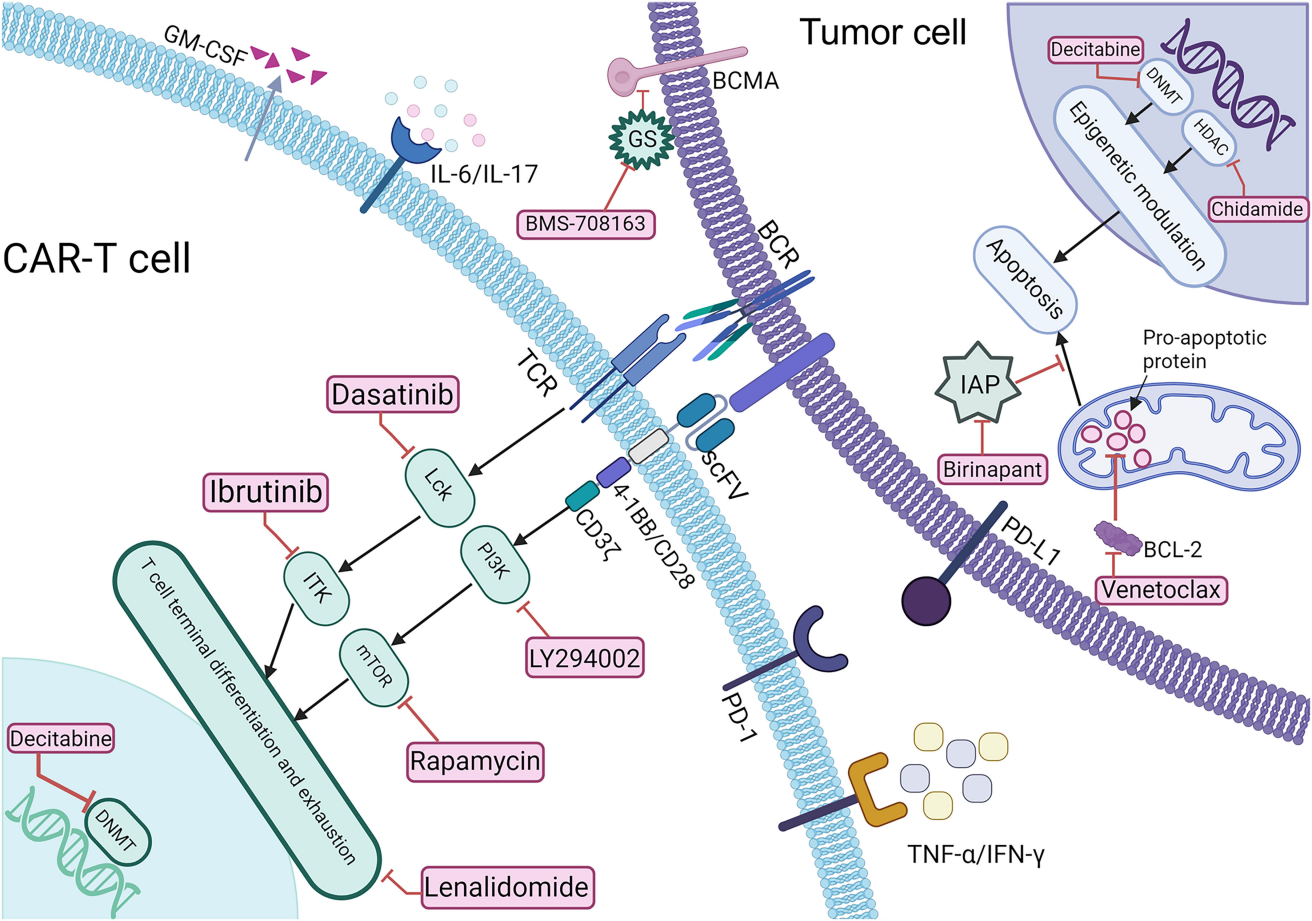
Schematic representation of the mechanisms underlying CAR-T therapy combined with Small-molecule reagents. BCR, B cell receptor; sBCMA, soluble B cell maturation antigen; TCR, T cell receptor; BCL-2, anti-apoptotic protein; IAP, inhibitor of apoptosis protein; GS, γ-secretase; PD-1/PD-L1, programmed death-1/programmed death-ligand 1.

As depicted in **Figure 1**, various small-molecule inhibitors targeting key signaling pathways play crucial roles in cancer therapy. These agents can be used alone or combined with CAR-T immunotherapy, synergistically enhancing anti-tumor efficacy and safety by promoting CAR-T cell proliferation and reversing exhaustion, augmenting cytotoxicity (9, 10), remodeling the iTME (11), inducing tumor apoptosis (12), upregulating tumor antigen expression (13), and reducing treatment-related toxicities (14), thereby optimizing clinical outcomes in hematologic malignancies.

### 2.1 Bruton’s tyrosine kinase inhibitor

BTK inhibitor ibrutinib enhances the therapeutic efficacy of CAR-T therapy by inhibiting interleukin-2 (IL-2)-induced T cell kinase (ITK) signaling pathway, promoting Th1 immune responses, enhancing T cell expansion, and downregulating the exhaustion-related surface molecules such as TIM-3,LAG-3,and PD-1 (15–18). Ibrutinib has demonstrated significant clinical efficacy in hematologic malignancies, In the RESONATE-2 trial, first-line ibrutinib therapy for chronic lymphocytic leukemia (CLL) (19) achieved an overall response rate (ORR) of 91%, while in mantle cell lymphoma (MCL) patients, ORR was 66% (20), collectively highlighting the robust clinical efficacy of ibrutinib and supporting its combination with CAR-T therapy for relapsed/refractory B-cell malignancies.

### 2.2 Lck inhibitor

The Lck inhibitor precisely modulates CAR-T cell function through reversible suppression of Src family kinase signaling (e.g., Lck), and prolongs CAR-T cell persistence in preclinical studies (21). In mouse models, short-term dasatinib administration effectively prevents cytokine release syndrome (CRS), with CAR-T cell function rapidly restored upon drug withdrawal (22); building on this rationale, a single-center, two-arm phase I trial (NCT04603872) is investigating dasatinib in combination with CD19/BCMA-targeted CAR-T cells in patients with relapsed or refractory B-cell hematologic malignancies (planned enrollment of 120 patients, including B-cell acute lymphoblastic leukemia, B-cell non-Hodgkin lymphoma, and Multiple Myeloma). The trial is ongoing, and its clinical efficacy warrants further investigation.

### 2.3 PI3K/AKT/mTOR signaling pathway

PI3K/AKT/mTOR signaling pathway plays a central role in T cell metabolism, differentiation, and exhaustion (23). PI3K inhibitors, such as LY294002, have been shown to enhance CAR-T cell persistence (24) while the mTOR inhibitor rapamycin promotes CAR-T cell differentiation into a central memory phenotype, enhancing their bone marrow tissue-homing capacity within the hematopoietic niche (25). Additionally, PI3K inhibitor duvelisib has demonstrated potential for CRS prophylaxis following CAR-T cell therapy. In a Phase I trial by Michael Slade et al. (26). (NCT05044039), all 18 patients experienced only grade 1 (86%) or grade 2 (14%) CRS, with no grade 3–4 events. Among evaluable patients, the preliminary ORR was 72% (13/18), with a CR rate of 50%, suggesting that duvelisib is a well-tolerated and potentially efficacious strategy for CRS prevention.

### 2.4 BCL-2 inhibitor

BCL-2 inhibitor Venetoclax enhances tumor cell susceptibility to CAR-T cells by antagonizing BCL-2-mediated anti-apoptotic signaling and inducing G1-phase cell cycle arrest (27). In B-cell lymphoma models, combining Venetoclax with CD19 CAR-T cells significantly enhanced tumor cell apoptosis and improved CAR-T cell persistence, demonstrating its potential clinical application for patients harboring BCL-2-expressing leukemia and lymphoma (28). However, the potential clinical benefits require validation in further studies.

### 2.5 Inhibitor of apoptosis protein

Inhibitor of apoptosis protein (IAP) antagonists, such as Birinapant, could augment the cytotoxic function of CAR-T cells by alleviating IAP-mediated suppression of Caspase-3, -7, and -9, thereby activating the RIPK1-dependent apoptotic pathway (29). In leukemia models, coadministration of Birinapant with CD19 CAR-T significantly increased the apoptosis of tumor cell and showed promise in overcoming CAR-T therapy resistance (30). Additionally, Birinapant enhances CAR-T–mediated bystander cell death and activates the NF-κB signaling pathway, thereby potentiating CAR-T cell antitumor activity, as demonstrated in glioblastoma models (31). Although clinical evidence for combining IAP antagonists with CAR-T therapy in hematologic malignancies is currently lacking, these results provide a mechanistic rationale for their potential application in hematologic malignancies.

### 2.6 Epigenetic modulators

Epigenetic modulators enhance antitumor activity by regulating DNA methylation, histone modifications, and chromatin remodeling, thereby upregulating tumor-associated antigen expression and optimizing CAR-T cell function. For instance, DNA methyltransferase inhibitors (DNMTis), such as decitabine (DAC), could restore tumor-associated antigen expression via promoter demethylation-mediated epigenetic reprogramming, thus enhancing CD19 CAR-T cell recognition and cytolytic activity (32); Changju Qu et al (33). investigated the efficacy and safety of decitabine-primed CD19/CD20 CAR-T therapy in R/R DLBCL. Among 16 patients, the CR rate was 63.6% and the ORR was 90.9%; grade 3 CRS occurred in 21.2% and ICANS in 9.2%. These findings suggest that DNMTi combined with CAR-T therapy is a promising approach, meriting further clinical evaluation to confirm its efficacy and safety.

### 2.7 Histone deacetylase inhibitors

Histone deacetylase inhibitors (HDACis), such as chidamide, enhance CAR-T cell efficacy by upregulating CD22 antigen density and alleviating T-cell exhaustion via modulation of the HDAC1–H3K27ac axis and activation of Wnt/β-catenin signaling (10). Moreover, valproic acid has been shown to increase CD20 expression, and Preclinical studies have demonstrated that treatment of B-cell malignancies with HDACi induces H3K9 acetylation at the CD20 gene promoter, leading to upregulation of CD20 expression. This epigenetic modulation substantially augments CAR-T cell cytotoxicity (34). These findings provide a rationale for investigating HDACi in combination with CAR-T therapy in hematologic malignancies.

### 2.8 Immunomodulatory reagents

Immunomodulatory reagents, including lenalidomide (LEN), potentiate antitumor responses by inducing Th1-skewed immune responses, enhancing T cell activation, and promoting CAR-T cell memory phenotype and longevity (35). Notably, Lenalidomide has demonstrated therapeutic efficacy in multiple myeloma (MM) and diffuse large B-cell lymphoma (DLBCL) patients, leading to the improved objective response rates and prolonged progression-free survival (36, 37). Guoxing Zhao et al. (38). reported a case of RRMM, offering preliminary clinical evidence for the feasibility and efficacy of combining anti-BCMA CAR-T cell therapy with lenalidomide. The patient, who relapsed after treatment with immunomodulatory drugs and proteasome inhibitors, underwent BCMA CAR-T cell infusion, achieving a very good partial response (VGPR) that was maintained for over eight months. This case provides a rationale for combining CAR-T therapy with lenalidomide as a potentially promising approach to improve outcomes in RRMM, although its efficacy and safety require validation in larger clinical studies.

### 2.9 Janus kinase inhibitors

Janus kinase (JAK) inhibitors, such as ruxolitinib, attenuate CRS-related inflammatory responses by inhibiting JAK-STAT signaling pathways (39). In preclinical CRS models, itacitinib (JAK1 inhibitor) significantly reduced the release of inflammatory cytokines, including interferon-γ (IFN-γ) and interleukin-6 (IL-6), IL-12, highlighting its potential in mitigating CRS (40). However, its therapeutic potential requires confirmation in larger, prospective studies.

### 2.10 γ-Secretase inhibitors

γ-Secretase inhibitors (GSIs) potentiate the cytolytic activity of BCMA CAR-T cells by preventing BCMA proteolytic cleavage, thereby upregulating BCMA surface density on tumor cells. In a phase I clinical trial for relapsed/refractory multiple myeloma (R/R MM), GSIs have exhibited a favorable safety and efficacy profile (41). In this study, 18 patients received BCMA CAR-T cells following a three-day crenigacestat preconditioning. The sCR rate was 44% and ORR 89%. CRS occurred in 94% of patients (grade 1: 50%), and ICANS in 39%. Given the limited sample size, the efficacy and safety of this approach warrant further validation in larger clinical trials.

Future optimization of CAR-T therapy requires rational integration of small-molecule reagents, informed by comprehensive pharmacokinetic/pharmacodynamic (PK/PD) modeling and systematic characterization of key signaling pathways. This strategy will enable the rational design of dosing schedules, optimized drug sequencing, and refined combination regimens, aiming to enhance the therapeutic efficacy while minimizing adverse effects, thereby facilitating the clinical translation of CAR-T therapies for relapsed/refractory hematologic malignancies.

## 3 Assessment of the clinical efficacy and safety profile of combination regimens

### 3.1 Diffuse large B-cell lymphoma

DLBCL, accounting for 30%-40% of non-Hodgkin lymphoma cases (42), predominantly affects older adults, with fewer than 5% of cases occurring in pediatric patients. Despite R-CHOP regimen serving as the standard first-line therapy, approximately 40% of adults exhibit primary resistance or early relapse events, leading to a poor 5-year overall survival (OS) rate (<10%) in the relapsed/refractory (R/R) setting (43). Mechanisms of resistance to R-CHOP regimen mainly including constitutive activation of the NF-κB pathway, TP53 inactivating mutations, and the inherent limitations of anti-CD19 CAR-T therapy (44). While anti-CD19 CAR-T therapy achieves an objective response rate (ORR) of 50%-60%, but long-term complete remission (CR) is achieved only in 30%-40% of patients, with 20%-30% developing progressive disease (PD) due to CD19 antigen escape (7, 45). These challenges highlight the increasing interest in rational combination strategies that integrate CAR-T therapy with small-molecule inhibitors for R/R DLBCL (**Table 1**). Dong et al. (46), conducted a multi-center retrospective cohort study comparing ibrutinib combined with CD19-targeted CAR-T cell immunotherapy (n=16) versus CAR-T monotherapy (n=10) in patients with R/R DLBCL. The combination arm demonstrated superior efficacy: complete response (CR) rate 43.7% (7/16) *vs*. 30.0% (3/10), partial response (PR) 43.7% (7/16) *vs*. 10.0% (1/10), resulting in a significantly higher objective response rate (ORR 87.5% [14/16] *vs*. 40.0% [4/10]; p<0.01). Safety analyses revealed that CRS incidence of 73.1% overall (19/26), with grade ≥2 CRS occurring in 2 patients in CAR-T monotherapy arm (20.0%) *vs* 4 patients in combination arm (25.0%). ICANS was observed exclusively in the combination group (12.5% [2/16]). Hematologic toxicities In the combination therapy group were neutropenia (6.2%[1/16]) and thrombocytopenia (6.2%[1/16]), along with one case of pulmonary infection (6.2%[1/16]). In the control group, neutropenia was observed in 2 patients (20%) and urinary tract infection in 1 patient (10%), with no thrombocytopenia observed. This study indicates that the addition of ibrutinib to CAR-T therapy may improve short-term remission rates in patients with R/R DLBCL; however, the limited sample size and retrospective nature of the analysis necessitate confirmation in prospective studies, and the potential toxicities warrant cautious evaluation. Changju Qu et al. (33) conducted a phase II clinical trial to assess the therapeutic potential and associated toxicities of CD19/CD22-targeted CAR-T therapy combined with a decitabine-based lymphodepleting regimen in R/R DLBCL patients. Thirty-three patients received the combination of decitabine, fludarabine, and cyclophosphamide (DFC), followed by CAR-T therapy. The results demonstrated CR rate of 63.6%, PR rate of 27.3%, and ORR of 90.9%. The median follow-up was 10.9 months, with PFS of 10.2 months, the PFS and 2-year OS rates were 47.2% and 54.3%, respectively, no grade 4 CRS was observed, and the incidence of grade 3 CRS was 21.2% (7/33), three patients developed mild ICANS. The most frequent adverse events were grade 3/4 hematologic toxicities, including neutropenia (100%), severe anemia (69.7% [23/33]), thrombocytopenia (90.9% [30/33]), and coagulopathy (36.4% [12/33]). Non-hematological and non-neurological adverse events included fever (78.8%), hypoxia (36.4%), hypotension (3.0%), heart failure (6.0%), and acute kidney injury. These results indicate that CD19/CD22 dual-targeted CAR-T therapy with a decitabine-based regimen shows preliminary efficacy in R/R DLBCL; however, in the absence of a control group, with significant hematologic and systemic toxicities and limited follow-up, its efficacy and safety must be validated in larger randomized trials. Moreover, Nana Ping et al. (37) conducted a prospective cohort study evaluating lenalidomide maintenance therapy following CAR-T treatment in R/R DLBCL. Among patients receiving maintenance therapy (n = 7), lenalidomide was administered at an initial dose of 25 mg/day on a 21-day-on, 7-day-off schedule within a 28-day cycle, continued until disease progression or relapse, with dose adjustments or temporary interruptions implemented based on hematologic parameters and treatment-emergent adverse events. The ORR in this group was 85.7% (6/7; 3 CR, 3 PR) versus 77.8% (7/9; 3 CR, 4 PR) in the observation group (p=0.65). Safety analyses revealed a comparable incidence of grade ≥3 CRS (42.9% [3/7] *vs*. 44.4% [4/9]; p=0.92), with reduced hematologic toxicity in the lenalidomide arm: grade 3/4 anemia (hemoglobin <8 g/dL) 14.3% (1/7) *vs*. 55.6% (5/9; p=0.04) and thrombocytopenia (platelet count <50×10^9^/L) 14.3% (1/7) *vs*. 44.4% (4/9; p=0.16), while neutropenia rates (absolute neutrophil count [ANC] <1.0×10^9^/L) remained comparable (71.4% *vs*. 77.8%). Similarly, lenalidomide maintenance may slightly improve short-term remission in R/R DLBCL and, through its immunomodulatory effects, mitigate excessive immune activation induced by CAR-T cells, thereby reducing hematologic toxicities associated with immune-related adverse events, including CRS. However, these potential benefits require confirmation in larger studies.

**Table 1:** Efficacy and safety of CAR-T therapy combined with targeted small-molecule inhibitors in R/R DLBCL.

Reference	Combination drugs	Clinical trial	Efficacy (including CR, ORR, PFS, and OS)	Adverse events
Lin Dong et al. (46)	Ibrutinib	NCT02537977	CR: Experimental:43.7% vs. Control:30.0% ORR: Experimental:87.5% vs. Control:40%	Ibrutinib group: Grade 2 CRS 25%, Grade 1 CRS 50%; Neurological symptoms 12.5%. Control group: Grade 2 CRS 20%, Grade 1 CRS 50%.
Yan Lu et al. (47)	Zanubrutinib	NCT02537977	CR:52.4%; ORR:81% 1-year PFS: 52.4%; 1-year OS: 80.1%	Grade 2 CRS 57.1%, Grade 1 CRS 28.6%; Grade 1 ICANS 9.5%; Grade 4 neutropenia 52.4%, thrombocytopenia 33.3%.
Changju Qu et al. (33)	Decitabine	NCT03196830	CR:63.6%; ORR:90.9% 2-year PFS: 47.2% 2-year OS: 54.3%	Grade 3 CRS 21.2%, Grade 2 CRS 12.1%, Grade 1 CRS 42.4%; ICANS 9.1% (co-occurring with severe CRS); Grade III/IV neutropenia100%; Grade III/IV thrombocytopenia 90.9%.
Nana Ping et al. (37)	Lenalidomide	Retrospective observational study	CR:42.8% Experimental:85.7% vs. Control:77.8%	Lenalidomide group: Grade III/IV CRS 42.9%, Leukopenia71.4%. Control group: Grade III/IV CRS 44.4%, Leukopenia 77.8%.
Chenggong Li et al. (48)	Lenalidomide	One case report	complete metabolic response (CMR) over 24 months	Grade 2 CRS; Grade 3–4 hematologic toxicity. No severe neurotoxicity reported.

Overall, combination regimens integrating targeted small-molecule inhibitors with CAR-T therapy have shown significant antitumor activity in R/R DLBCL (**Table 1**). with distinct profiles: Ibrutinib primarily enhances partial responses with mild toxicity; Decitabine combined with dual-targeted CAR-T achieves higher initial complete responses but induces significant hematologic and systemic toxicities; and lenalidomide maintenance sustains responses while mitigating some toxicities. Nonetheless, the treatment-related toxicities, particularly CRS and ICANS, remain major barriers to clinical application. Some combinations have failed to reduce the toxicity and may even exacerbate adverse effects, thereby limiting their clinical feasibility. Moreover, existing studies are small, non-randomized, and of limited follow-up, leaving long-term efficacy and safety uncertain. Future research should focus on drug screening, optimization of the dosing schedule, and dose refinement to enhance the therapeutic efficacy while minimizing toxicity. Additionally, further translational and clinical studies are essential to identify the most effective combinatorial strategies, refine patient selection criteria, and mitigate treatment-related toxicities, ultimately facilitating the clinical translation and wider clinical adoption of this strategy.

### 3.2 Chronic lymphocytic leukemia

CLL, the most prevalent adults leukemia in western, accounts for approximately 25%–30% of all leukemia cases. The median age at diagnosis is approximately 70 years, and the disease is exceptionally rare in pediatric patients. Approximately 30%–40% of CLL patients harbor high-risk genetic abnormalities, such as del(17p)/TP53 mutation and unmutated immunoglobulin heavy-chain variable region (IGHV), both of which are associated with the adverse clinical outcomes and a 5-year OS rate of less than 30% (49, 50). Conventional chemoimmunotherapy regimens, such as fludarabine-cyclophosphamide (FC), demonstrate limited efficacy in patients with TP53 aberrations or complex karyotypes (≥3 chromosomal abnormalities), with CR rates less than 10% (51). In the R/R CLL setting, CD19-targeted CAR-T therapy achieves an ORR of 50%–70%; however, the rate of durable CR remains low, at only 20%–30% (52). The iTME, characterized by high PD-L1 expression, T-cell exhaustion, and CD19 antigen loss—observed in 10%–20% of relapsed cases—contributes to the therapeutic resistance via multiple immune escape mechanisms (53, 54). Emerging evidence indicates that combining CAR-T therapy with small-molecule inhibitors can mitigate TME-induced immunosuppression and antigen escape, ultimately leading to improved long-term disease control. Jordan Gauthier et al. (55) conducted a phase I/II non-randomized clinical trial (NCT01865617) to evaluate the feasibility, safety, and efficacy of CD19-targeted CAR-T therapy combined with ibrutinib in patients with R/R CLL refractory to ibrutinib monotherapy. All patients had received at least one prior ibrutinib-containing regimen. In the experimental group, patients initiated daily ibrutinib at 420 mg at least two weeks prior to CAR-T infusion and continued therapy for a minimum of three months post-infusion. The control group comprised contemporaneous R/R CLL patients who received CD19-targeted CAR-T cell therapy alone, with ibrutinib discontinued prior to CAR-T infusion and not resumed during treatment. Given the non-randomized design, selection bias in the control group cannot be excluded. The study also assessed the potential role of ibrutinib co-administration in mitigating CRS severity. At week 4, the ORR in the combination therapy group was 83%, with a CR rate of 22% and a PR rate of 61%, whereas the ORR in the control group was 56%. Bone marrow MRD negativity was achieved in 61% and 72% of patients, as determined by flow cytometry and immunoglobulin heavy chain (IGH) sequencing. The 1-year OS and PFS rates were 64% and 38% in the combination therapy group. Among patients with MRD negativity, the OS rates and 1-year PFS were 86% and 59%, respectively, these results showing a trend toward improved survival compared with the control group (P = 0.09). The median CRS grade was 1 (range: 0–2) in the combination therapy group, significantly lower than 2 (range: 0–5) in the control group (P = 0.04). The incidence (26% *vs*. 42%) and severity of ICANS were comparable between two groups. Seven patients in the combination therapy group developed ibrutinib-related adverse events, necessitating dose reduction or discontinuation in six patients (32%), while one patient died from suspected ibrutinib-associated arrhythmia. Grade ≥3 neutropenia was observed in all patients, while anemia and thrombocytopenia occurred in 79% and 68% of patients, respectively, with most cases being transient. Additionally, one case of pulmonary embolism and one case of microembolic stroke were reported. Similarly, Saar Gill et al. (56) conducted a prospective, single-center, phase II trial (NCT 02640209) to investigate the efficacy, safety, and durability of CD19 CAR-T therapy combined with ibrutinib in patients with CLL. The study enrolled 19 patients, with a median follow-up of 41 months (range: 0.25–58 months), at 3 months, the CR rate, as defined by the International CLL Working Group (iwCLL), was 44%, 72% patients achieved undetectable MRD at 12 months, the OS rate was 84% and the PFS rate was 70% at 48 months, regarding the adverse events, 53% patients experienced grade 3–4 neutropenia and 37% experienced grade 3–4 thrombocytopenia, CRS occurred in 18 patients, including one case of grade 4 CRS and grade 4 ICANS, resulting in death on day 10 post-infusion. Cameron J. Turtle et al. (57) conducted a phase I/II open-label trial to evaluate the safety, feasibility and efficacy of CD19 CAR-T therapy in ibrutinib R/R CLL patients. The study enrolled 24 patients, with an ORR rate of 71%, among 19 patients with imaging-based response assessment at week 4 post-infusion, the ORR was 74%, comprising a CR rate of 21% and a PR rate of 53%, regarding the adverse events, CRS occurred in 83% patients, while ICANS was observed in 33% patients, importantly, all ICANS cases were observed in patients who also experienced CRS. In addition to evaluating the combination of ibrutinib and CAR-T therapy in R/R CLL, this study also investigated the potential role of incorporating phosphoinositide 3-kinase δ/γ (PI3Kδ/γ) inhibitors, duvelisib, during CAR-T cell manufacturing, with 300 nM duvelisib added to the culture medium every three days. this approach aimed to induce epigenetic and metabolic reprogramming of CAR-T cells to enhance their antitumor activity and improve therapeutic outcomes in CLL.The experimental group (Duv-CART) consisted of CAR-T cells treated with duvelisib, whereas the control group comprised conventionally manufactured CAR-T cells. Compared with controls, Duv-CART cells demonstrated significantly enhanced *in vitro* cytotoxicity against CD19^+^ CLL targets and prolonged survival in CLL-bearing mouse models. These preliminary results suggest a potential therapeutic advantage of duvelisib incorporation, although its clinical efficacy requires further validation (58).

Overall, the combination of ibrutinib and CAR-T therapy demonstrates significant therapeutic efficacy in R/R CLL (**Table 2**). This holds true whether CAR-T therapy is administered concurrently with ibrutinib or used as monotherapy following ibrutinib failure, both of which result in favorable clinical responses. Additionally, this combination approach may help mitigate the severity of CRS and reduce the incidence of neurotoxicity, although it does not substantially improve hematologic toxicity. Future studies should focus on optimizing the combination regimen, determining the optimal dosing and administration schedule, and exploring broader small-molecule inhibitor combinations to enhance both efficacy and safety.

**Table 2:** Efficacy and safety of CAR-T therapy combined with targeted small-molecule inhibitors in R/R CLL.

Reference	Combination drug	Clinical trial	Efficacy (including CR, ORR, PFS, and OS)	Adverse events
William G. Wierda et al. (59)	Ibrutinib	NCT03331198	CR:47%; ORR:95%	CRS: 74%, Grade 3 CRS: 5.2%; Neurological symptoms: 32%; Grade ≥3 neutropenia: 89%, anemia: 47%.
Cameron J. Turtle et al. (57)	Ibrutinib	NCT01865617	CR:21%(4/19); ORR:71%(17/24), 74%(14/19)	Grade 5 CRS: 4%, Grade 4 CRS: 4%, Grade 1–2 CRS: 75%; Neurotoxicity: 33% (Grade 5: 4%, Grade 3: 20.8%, Grade 1-2: 8.3%).
Saar Gill et al. (56)	Ibrutinib	NCT02640209	CR:44%(at 3 months); 48-month PFS: 70%,48-month OS: 84%	CRS: 94.7% (Grade 1-2: 78.9%); Neurotoxicity: 26.3%, including 1 fatal case due to Grade 4 CRS and ICANS; Grade 3–4 neutropenia: 53%, thrombocytopenia: 37%.
Tanya Siddiqi et al. (60)	Ibrutinib	NCT03331198	CR:45%; ORR:82%	CRS: 74%, Grade 3 CRS: 9%; Neurological events: 39% (Grade 3-4: 22%); Serious adverse events (SAEs): 57%; Mortality rate: 40.9%; Neutropenia: 70%, anemia: 83%, thrombocytopenia: 74%.
Jordan Gauthier et al. (55)	Ibrutinib	NCT01865617	CR:22%; ORR: Treatment group: 83% *vs*. Control group: 56%; 1-year PFS: Treatment group:38%*vs*. Control group:50%; 1-year OS: Treatment group:64%*vs*. Control group: 61%	Combination group: Median CRS Grade 1 (range 0-2), neurotoxicity: 26%. Control group: Median CRS Grade 2 (range 0-5), neurotoxicity: 42%. Grade ≥3 neutropenia:100%, thrombocytopenia: 68%, anemia: 79%.

### 3.3 Multiple myeloma

MM is a plasma cell malignancy accounting for 10%–15% of all hematologic malignancies. The disease primarily affects individuals aged more than 60 years old, with a median age at diagnosis of 65–70 years. Therapeutic advances, including anti-CD38 monoclonal antibodies, proteasome inhibitors (PIs), and immunomodulatory drugs (IMiDs), have markedly improved patient survival (61) (62), however, almost all patients eventually develop RRMM, leading to a substantial decline in the 5-year OS (63), which is often less than 30%. BCMA-directed CAR-T therapy induces an ORR of 70%–90% in RRMM (64), while the rate of sustained CR remains below 50% (65). BCMA antigen escape represents a key mechanism of disease relapse following CAR-T therapy, occurring in approximately 30%–50% of cases. Moreover, T-cell exhaustion and the iTME compromise the durability of CAR-T cell responses (66) (67). Emerging evidence suggests that combining CAR-T therapy with small-molecule inhibitors may enhance T-cell function and modulate the TME, thereby mitigating antigen escape and enhancing response durability. Shi Xiaolan et al (68), conducted a single-center, phase II clinical trial (NCT03455972) to evaluate the safety and efficacy of anti-CD19/BCMA dual-targeted CAR-T therapy combined with lenalidomide maintenance following autologous stem cell transplantation (ASCT) in patients with high-risk newly diagnosed multiple myeloma (NDMM). The study enrolled 10 patients, achieving an ORR of 100%, with 90% (n=9) attaining a sCR and 10% (n=1) achieving CR. At the time of analysis, the median PFS and OS had not yet been reached. MRD negativity was observed in 50% of patients within 100 days post-ASCT, and at the 42-month follow-up, 70% maintained MRD negativity for over 24 months. All patients developed CRS of varying grades. Grade ≥3 hematologic toxicities were reported, including lymphocytopenia (100%), neutropenia (20%), anemia (50%), and thrombocytopenia (70%), all of which resolved within one month following supportive care. Infectious complications included five cases of upper respiratory tract infections. Non-hematologic adverse events consisted of transient hyperbilirubinemia (n=1) and hypotension (n=1). Importantly, no cases of ICANS or treatment-related mortality were reported. Similarly, Guoxing Zhao et al. (38) reported a case of RRMM treated with BCMA-directed CAR-T therapy in combination with lenalidomide. The patient achieved a VGPR at day 14 post-infusion, with bone marrow plasma cell infiltration decreasing to 0.08% (95% CI: 0.02–0.15%) and confirmed MRD negativity by next-generation flow cytometry. This response persisted for an 8-month duration until disease progression at the 9-month follow-up, characterized by reappearance of serum M-protein, elevated λ free light chain (FLC) levels, and increased plasma cell infiltration (4.5% in bone marrow biopsy). During the treatment course, the patient experienced grade 3 myelosuppression. After the second CAR-T infusion, grade 2 CRS occurred, but no neurotoxicity was observed. Collectively, CAR-T therapy combined with lenalidomide induces rapid, deep responses and short-term MRD negativity in high-risk NDMM and RRMM, but responses are often transient, relapse is frequent, and reversible hematologic toxicities and CRS remain prevalent. Furthermore, Andrew J. Cowan et al. (41) conducted a single-arm, phase I dose-escalation study to evaluate the safety and pharmacodynamic interaction of γ-secretase inhibitors (GSI) combined with BCMA-targeted CAR-T therapy in R/R MM. The study aimed to investigate whether GSI could enhance BCMA antigen density, thereby potentiating CAR-T cell cytotoxicity. Eighteen patients were enrolled, with the best overall response being sCR in 44% (8/18), CR in 11% (2/18), and VGPR in 22% (4/18), resulting in an ORR of 89%. The median PFS was 11 months, and OS was 42 months, with a median follow-up of 42 months. In terms of adverse events, CRS were observed in 94% of patients, with 50% of cases being grade 1 and 33% grade 2. ICANS was observed in 39% of patients, primarily of grade 1–2. Notably, 94% of participants experienced ≥grade 3 adverse events. Thus, while GSI combined with BCMA-targeted CAR-T markedly improves overall response rates in R/R MM, the high incidence of ≥3-grade toxicities warrants cautious safety evaluation.

Overall, the combination of small-molecule inhibitors and CAR-T therapy shows substantial therapeutic potential in RRMM (**Table 3**). However, safety concerns remain, as adverse events such as CRS and bone marrow suppression continue to pose significant clinical challenges. Lenalidomide combined with CAR-T demonstrates notable efficacy with manageable toxicity, albeit with limited durability, whereas GSI plus CAR-T further elevates overall response rates but incurs considerable high-grade toxicity, underscoring the need to balance efficacy and safety. Further research is warranted to explore the strategies for mitigating these adverse effects while preserving therapeutic efficacy.

**Table 3:** Efficacy and safety of CAR-T therapy combined with targeted small-molecule inhibitors in R/R MM.

Reference	Combination drug	Clinical trial	Efficacy (including CR, ORR, PFS, and OS)	Adverse events
Xiaolan Shi et al. (68)	Lenalidomide	NCT03455972	sCR90%, CR10%; ORR:100% Median PFS and OS not reached	Grade 2 CRS 50%, Grade 1 CRS 50%; No neurotoxicity; ≥Grade 3 lymphopenia 100%, ≥Grade 3 respiratory infections 50%.
Guoxing Zhao et al. (38)	Lenalidomide	One case report	VGPR over 8 months; bone marrow plasma cells decreased to 0.08% with MRD negativity.	Grade 2 CRS occurred after the second CAR-T infusion, lasting 7 days; Grade 3 myelosuppression led to lenalidomide discontinuation; IL-6 peak level: 119.8 pg/mL, transient elevation of inflammatory cytokines
Andrew Jcowan et al. (41)	γ-Secretase Inhibitor	NCT03502577	sCR 44%, CR 11%; ORR:89%; Median PFS: 11 months; Median OS: 42 months	CRS (94%): Grade 1 (50%), Grade 2 (33%); ICANS (39%), mostly Grade 1-2; ≥Grade 3 adverse events reported in 94% of participants

### 3.4 Other hematologic malignancies

The combination of CAR-T therapy and small-molecule inhibitors has also shown promising clinical efficacy in other hematologic malignancies. For instance, in patients with R/R MCL who have previously received small-molecule inhibitors, CD19-targeted CAR-T therapy has been reported to achieve an ORR of 80%–90%, with a long-term CR rate of 40%–50%. Nevertheless, key challenges remain, including CD19 antigen escape and T-cell exhaustion (69). M. Wang et al. (70), conducted a multicenter phase 2 trial to assess the efficacy of KTE-X19, a CD19-directed CAR-T cell therapy, in patients with R/R MCL. Unlike other CD19 CAR-T products, KTE-X19 incorporates optimized CD4+/CD8+ T cell ratios and a pre-infusion lymphodepletion regimen with cyclophosphamide and fludarabine to enhance *in vivo* expansion and persistence, thereby improving therapeutic outcomes. Sixty patients were enrolled, and the study reported an 93% ORR and a 67% CR rate. At 12 months, the OS rate was 83%, and the PFS rate was 61%. After a median follow-up of 12.3 months, 57% of patients remained in remission. At 24 months, 43% of patients remained progression-free, and 83% achieved MRD negativity by the fourth week. Treatment-related adverse events included CRS, observed in 91% patients, and neurotoxicity in 63% patients. Hematologic toxicities included ≥grade 3 neutropenia (85%), thrombocytopenia (51%), and anemia (50%). Additionally, 26% of patients experienced long-term cytopenia lasting more than 90 days, and 32% developed grade ≥3 infections. These results suggest that KTE-X19 demonstrates substantial efficacy in patients with MCL who have relapsed or refractory to BTK inhibitor therapy. However, it is associated with a considerable risk of toxicity, particularly CRS and neurotoxicity, necessitating close monitoring and proactive management.

Similarly, CD19-directed CAR-T therapy has emerged as a promising treatment approach for R/R B-cell acute lymphoblastic leukemia (R/R B-ALL), demonstrating deep and durable responses in pediatric patients. Moreover, CAR-T therapy has shown the improved efficacy in adult ALL, with 70%–90% CR rates (71, 72). Small-molecule inhibitors have been found to enhance the therapeutic efficacy of CAR-T therapy in R/R B-ALL through multiple mechanisms, including enhancing apoptotic signaling, modulating metabolic pathways, and potentiating immune responses. Yunju Ma et al. (73), conducted a retrospective analysis to evaluate the efficacy of a lymphodepletion regimen consisting of decitabine (DAC), fludarabine (Flu), and cyclophosphamide (Cy) prior to CD19/CD22 bispecific CAR-T therapy in R/R B-ALL patients. The study included 26 R/R B-ALL patients, none of whom had achieved remission prior to lymphodepletion. After 28 days, no significant differences were observed between the DAC and control groups in CR and MRD-negative CR rates. However, the 3-year OS was 92.3% in the DAC group and 41.7% in the control group (P = 0.005). The 3-year leukemia-free survival (LFS) was 92.9% in the DAC group and 27.3% in the control group (P < 0.001). The relapse rate among non-transplanted patients was 16.7% in the DAC group, compared to 75% in the control group. No significant difference was observed in the incidence of CRS between the two groups. The study suggests that DAC, as part of the lymphodepletion regimen, enhances the long-term efficacy of CAR-T therapy with an acceptable safety profile; however, further validation through large-scale randomized controlled trials is needed.

Overall, combination regimens incorporating small-molecule reagents and CAR-T cells exhibit higher response rates and improved long-term survival in R/R MCL and R/R B-ALL (**Table 4**). However, treatment-related toxicities such as CRS and ICANS remain significant challenges. Future research should focus on optimizing pretreatment strategies, minimizing treatment-related toxicities to enhance the safety and clinical applicability of these combination therapies.

**Table 4:** Efficacy and safety of CAR-T therapy combined with targeted small-molecule inhibitors in R/R MCL and R/R B-ALL.

Reference	Combination drug	Clinical trial	Efficacy (including CR, ORR, PFS, and OS)	Adverse events
Charles Herbaux et al. (74)	KTE-X19	Retrospective observational study	CR:61.9%; ORR:88%;6-month PFS: 57.9%	CRS: 78.7% (≥Grade 3: 8.5%); Neurotoxicity:48.9% (≥Grade 3: 8.5%); ICU admission: 27.7%; Mortality: 11.9% (4 due to disease progression, 1 due to Grade 5 CRS).
M. Wang et al. (70)	KTE-X19	NCT02601313	CR:67%; ORR:93%; 1-year PFS: 61%,1-year OS: 83%	CRS:91%(≥Grade3:15%); Neurotoxicity: 63%(≥Grade3:31%); ≥Grade3.neutropenia: 85%; ≥Grade 3 thrombocytopenia: 51%; ≥Grade 3 infections: 32%
Michael Wang et al. (75)	liso-cel	NCT02631044	CR:72.3%; ORR:83.1%; Median PFS: 15.3 months; Median OS: 18.2 months; Median OS for CR patients: 36.3 months.	CRS: 61%; Neurotoxicity: 31%; ≥Grade 3 neutropenia:56%; ≥Grade 3 anemia: 37.5%
Yunju Ma et al. (73)	Decitabine	NCT03614858	3-year OS: DAC group 92.3% vs. control 41.7%; 3-year LFS: DAC group 92.9% *vs*. control 27.3%	No significant difference in CRS incidence between DAC and control groups; Similar hematologic recovery times; All adverse events were reversible

## 4 Future strategies for enhancing CAR-T cell therapy

Therapy resistance and disease relapse remain the significant obstacles to CAR-T therapy. Small-molecule reagents have emerged as promising adjuncts, capable of alleviating T cell exhaustion, promoting the expansion of naïve and memory T cells, enhancing tumor-associated antigen (TAA) presentation, modulating the TME, and regulating CAR-T cell cytotoxicity and cytokine secretion. These mechanisms collectively enhance CAR-T cell therapeutic efficacy, contribute to the reduction of treatment-related toxicities, and significantly improve patient remission rates and overall survival. However, several challenges persist in clinical application. First, the complexity and heterogeneity of the TME, including immunosuppressive factors, metabolic reprogramming and diverse cellular components, may compromise the efficacy of combinatorial approaches, resulting in suboptimal therapeutic outcomes. Second, combination strategies may induce increased toxicities, such as exacerbating CRS and ICANS, which may lead to life-threatening complications. To overcome these limitations, the identification and innovation of small-molecule reagents, as well as the refinement and optimization of combinatorial approaches, may be the key strategies to enhancing the clinical success of CAR-T therapy.

**Figure 2:**
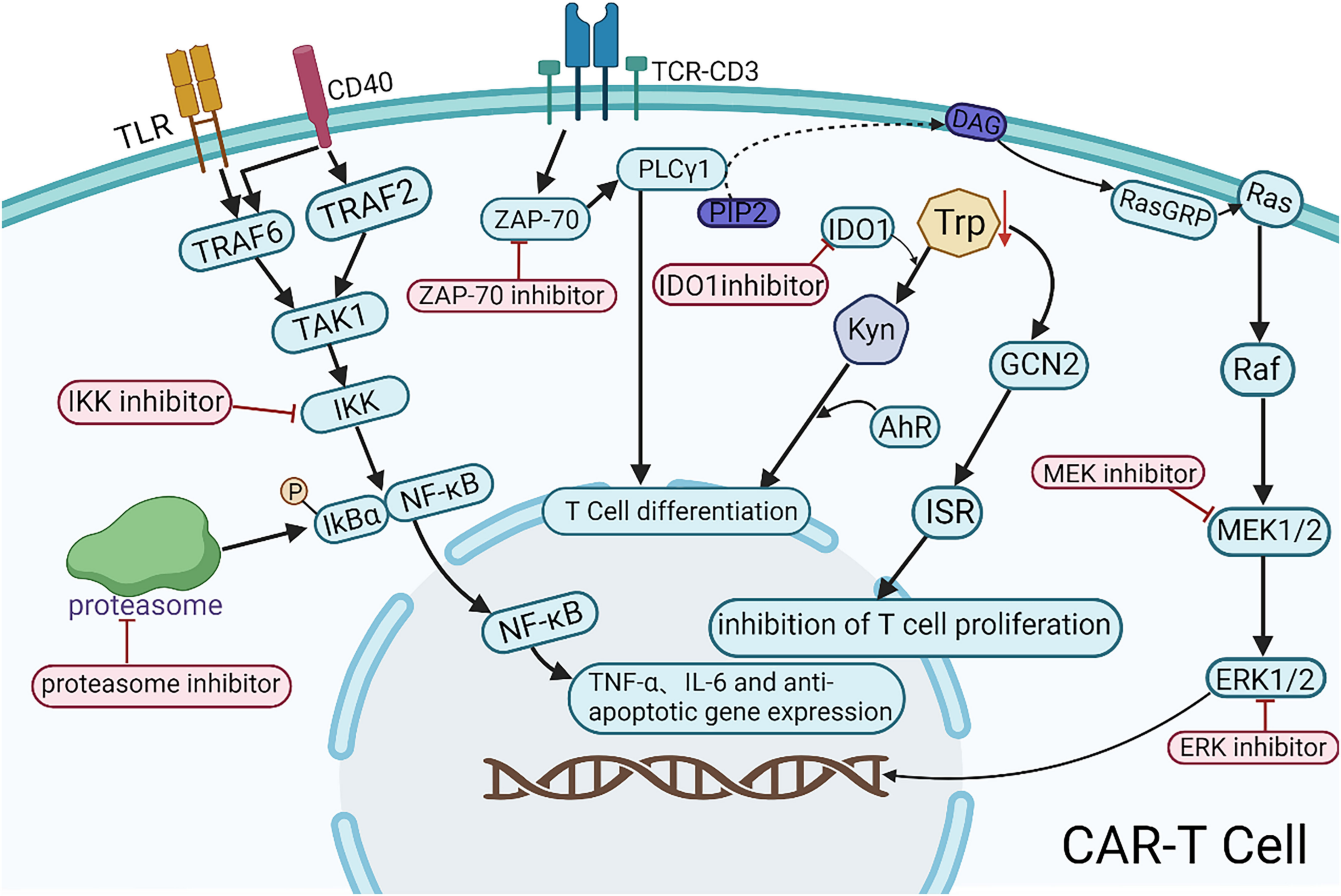
Mechanistic illustration of emerging small-molecule agents in combination with CAR-T therapy. TLR, a pattern recognition receptor; CD40, a member of the TNF receptor superfamily; TCR-CD3, the T-cell receptor (TCR) complex; DAG, diacylglycerol; Trp, tryptophan; Kyn, kynurenine.

Emerging small-molecule inhibitors, including Zeta-chain-associated protein kinase 70 (ZAP-70) inhibitors, IKK inhibitors, proteasome inhibitors, IDO inhibitors, and MEK inhibitors, may enhance the efficacy of CAR-T therapy (**Figure 2**). ZAP-70 is a key molecule in the T cell receptor (TCR) signaling pathway. Its inhibitors target the ATP-binding pocket of ZAP-70, inhibiting the activation of downstream PLCγ1 and ultimately blocking T cell differentiation. Controlled application of ZAP-70 inhibitors during CAR-T cell production may limit the differentiation and thereby prolong antitumor activity. However, the precise mechanisms and clinical feasibility of this strategy warrant further preclinical and clinical validation. IKK inhibitors and proteasome inhibitors can suppress the nuclear translocation of NF-κB either directly or indirectly, leading to the downregulation of inflammatory cytokines (e.g., TNF-α, IL-6) and anti-apoptotic genes, thereby contributing to tumor microenvironment modulation. In the future, NF-κB-targeted interventions combined with CAR-T therapy may offer a novel directions for the precision treatment of hematologic malignancies. IDO1 (Indoleamine 2,3-dioxygenase 1) is overexpressed in various malignancies and is associated with poor prognosis. IDO1 catalyzes the kynurenine pathway of tryptophan catabolism, suppressing T cell function and inducing immune tolerance. IDO1 inhibitors block this pathway, enhancing antitumor immune responses. Chengtao Sun et al. (76) demonstrated that IDO1 inhibition downregulates MDM2 expression in DLBCL cells, activates the p53 signaling pathway, and induces cell cycle arrest and apoptosis, ultimately improving patient outcomes. Therefore, IDO1 inhibitors may enhance CAR-T cell responses by reducing DLBCL resistance. MEK inhibitors facilitate the expansion of stem cell-like memory T (Tscm) cells via metabolic reprogramming, delaying cell division and proliferation while preserving an undifferentiated state (77). Short-term inhibition of the MAPK signaling pathway during CAR-T cell production may help regulate CAR-T cell activity and attenuate CRS and other inflammatory responses associated with CAR-T therapy. These small-molecule inhibitors hold potential for enhancing CAR-T therapy efficacy and broadening its combinatorial treatment strategies. Further studies are required to elucidate their precise mechanisms, optimize the combination regimens, and assess their clinical feasibility.

**Figure 3:**
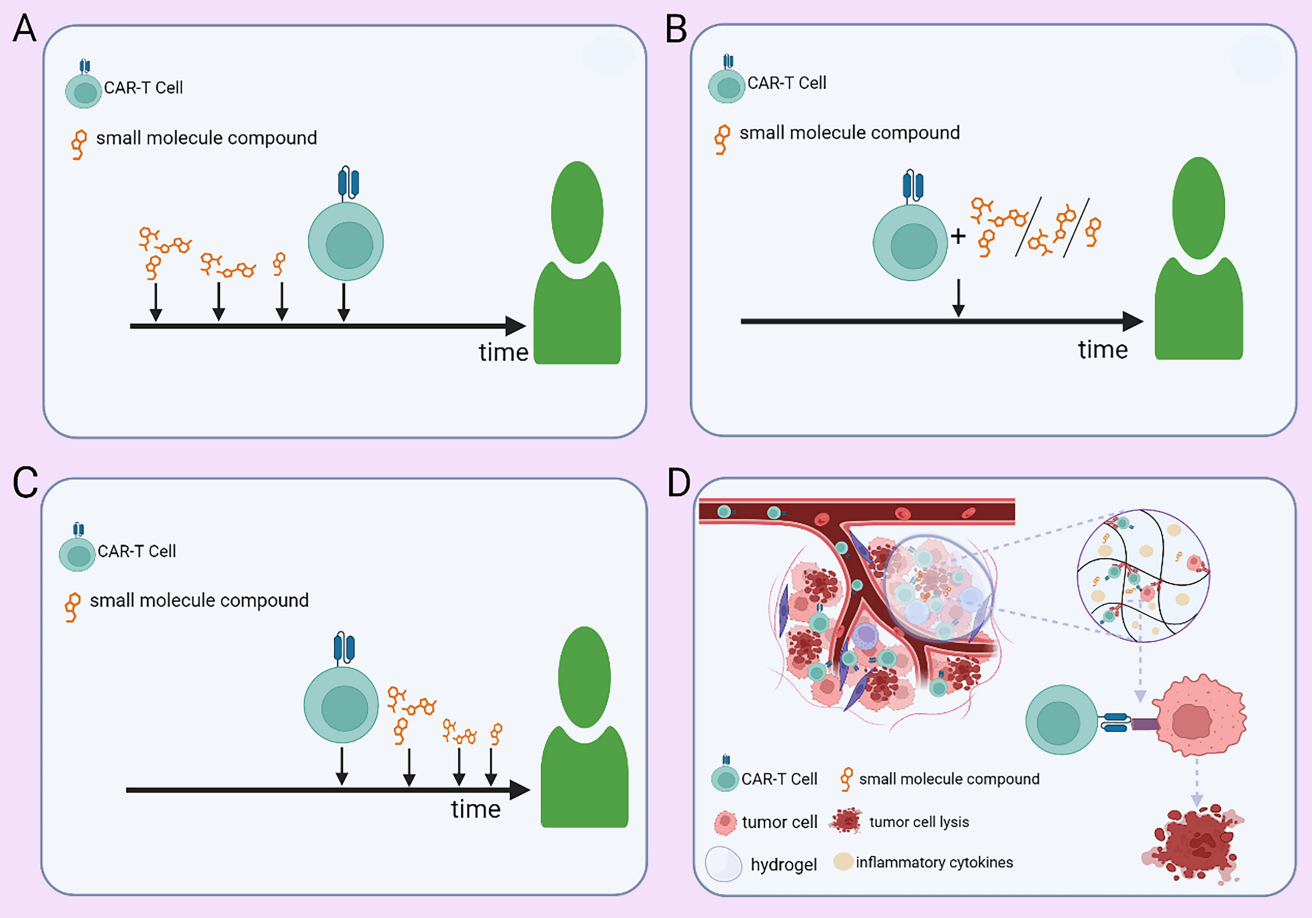
Strategies for administering small-molecule agents in combination with CAR-T therapy. **(A)** Preconditioning with small-molecule agents at optimized doses and time points before CAR-T infusion. **(B)** Concurrent administration of small-molecule agents at adjusted doses during CAR-T therapy. **(C)** Post-infusion maintenance therapy with small-molecule agents at predefined doses and time points. **(D)** Controlled co-delivery of CAR-T cells and small-molecule agents via a hydrogel-based sustained-release system within the tumor microenvironment.

Optimization of combination strategies for CAR-T therapy can be achieved through rational treatment scheduling, advanced delivery technologies, and the integration of additional therapeutic modalities. Optimizing the administration schedule of small-molecule inhibitors at different phases of CAR-T therapy (pre-infusion, peri-infusion, and post-infusion) is crucial for maximizing therapeutic efficacy (**Figures 3A–C**). Yong Gu Lee et al. (27) demonstrated that the BCL-2 inhibitor venetoclax promotes tumor cell apoptosis and enhances CAR-T efficacy *in vitro*. However, it concurrently exerts inhibitory effects on CAR-T cell function. Therefore, modulating its administration timing—such as pre-infusion dosing—may maximize therapeutic benefits while minimizing its inhibitory impact on CAR-T cell function. Nonetheless, further investigations are warranted to elucidate its mechanisms, optimize dosing regimens, and define the optimal dosing schedule for personalized combination therapy. In solid tumors, tumor-targeted nanoparticle-based delivery systems have the potential to enhance CAR-T cell homing to tumor sites, thereby increasing the precision of tumor eradication. Additionally, hydrogel-based controlled release allows for the sustained local delivery of CAR-T cells, prolonging their persistence within the TME and enhancing treatment durability (**Figure 3D**). In hematologic malignancies such as MM, nanoparticle-based drug delivery can promote preferential accumulation of small-molecule inhibitors within the TME, thereby enhancing intratumoral drug bioavailability while reducing systemic toxicity. Furthermore, hydrogel-based delivery platforms enable coordinated sustained release of both CAR-T cells and small-molecule inhibitors, enhancing their synergistic interactions while preventing the abrupt surge of CAR-T cells into circulation. This strategy may help dampen excessive immune activation and thereby mitigate the risk of CRS. Integrating nanomaterial-based technologies into CAR-T combination strategies may further refine therapeutic paradigms for hematologic malignancies and drive innovation in this field. Beyond conventional small-molecule and CAR-T combinations, incorporating additional therapeutic modalities may further enhance treatment efficacy. A leukemia model study by Saara Laukkanen et al. (78) demonstrated a significant synergistic effect between the mTORC1 inhibitor temsirolimus and the LCK inhibitor dasatinib. This suggests that short-term pre-infusion administration of temsirolimus may optimize T cell metabolic fitness and improve the long-term persistence of CAR-T cells, while transient dasatinib exposure during CAR-T therapy may fine-tune T cell activation, preventing exhaustion and excessive immune activation. Further investigation is required to ascertain whether this precisely timed combination can elicit a supra-additive effect beyond the expected summation of individual drug activities. In addition, preclinical and clinical studies are necessary to validate this approach and establish its therapeutic potential in hematologic malignancies.

## 5 Conclusion

The synergistic integration of small-molecule reagents with CAR-T therapy has demonstrated significant potential roles in enhancing both efficacy and safety. This review provides a comprehensive analysis of clinically investigated small-molecule reagents in combination with CAR-T therapy, emphasizing their mechanisms of action, therapeutic efficacy, and safety profiles. Furthermore, we explore the potential of emerging small-molecule reagents in combinatorial CAR-T strategies and proposed the evidence-based optimization approaches. While these combination therapies hold promise for improving clinical outcomes, rigorous preclinical validation and translational studies are essential to optimize efficacy and expand their applicability across diverse disease indications.
